# Largescale mullet (*Planiliza macrolepis*) can recover from thermal pollution-induced malformations

**DOI:** 10.1371/journal.pone.0208005

**Published:** 2018-11-29

**Authors:** Yi Ta Shao, Shang-Ying Chuang, Hao-Yi Chang, Yung-Che Tseng, Kwang-Tsao Shao

**Affiliations:** 1 Institute of Marine Biology, National Taiwan Ocean University, Keelung, Taiwan; 2 Center of Excellence for the Oceans, National Taiwan Ocean University, Keelung, Taiwan; 3 Marine Research Station, Institute of Cellular and Organismic Biology, Academia Sinica, Taipei, Taiwan; 4 Biodiversity Research Center, Academia Sinica, Taipei, Taiwan; Universite de Liege, BELGIUM

## Abstract

It is well known in aquaculture that hyperthermic perturbations may cause skeleton malformations in fish, but this phenomenon has rarely been documented in wild species. One rare location where thermal pollution has increased the proportion of malformed fish in wild population is in the waters near the Kuosheng Nuclear Power Plant in Taiwan. At this site, the threshold temperature and critical exposure time for inducing deformations have not been previously determined. In addition, it was unclear whether juvenile fish with thermal-induced malformations are able to recover when the temperature returns below the threshold. In the present study, juvenile largescale mullet (*Planiliza macrolepis*) were kept at temperatures ranging from 26°C and 36°C for 1–4 weeks, after which malformed fish were maintained at a preferred temperature of 26°C for another 8 weeks. The vertebrae bending index (VBI) of fish was increased after 2 weeks at 36°C, and deformed vertebral columns were detected by radiography after 4 weeks. However, malformations were not observed in groups kept at or below 34°C. Moreover, at the end of the recovery period, both the VBI and the vertebrae malformations had returned to normal. The results of this study may help to more precisely determine potential environmental impacts of thermal pollution and raise the possibility that the capacity for fish vertebrae to recover from the impacts of chronic thermal exposures may be an important consideration in marine fish conservation.

## Introduction

Human activities often create a large amount of waste that is discharged into nearby water bodies, where it affects resident organisms. While this waste often carries chemical byproducts and other artificial substances, xenobiotics are not the only pollutants to be found in discharge. Water with temperature that is relatively cooler or warmer than the receiving body can perturb the environment as well, and such thermal pollution may severely affect ecosystem composition and balance [1].

Thermal pollution is commonly caused by warm discharge from power plants and industrial manufacturers, or it may also arise from cold effluent at liquefied natural gas terminals. Acute exposure to temperature changes may cause thermal shock or mortality in fish or other aquatic organisms. Natural heat shock occurs at intertidal or thermal vent areas, while natural cold shock may occur when a cold water mass intrudes into coastal waters. Apart from acute lethality, thermal pollution has also been reported to alter physiological or behavioral characteristics of fish. For example, thermal perturbations were shown to influence the activity levels of carp (*Cyprinus carpio*) [2]. Physiological responses to thermal stress have also been found in several fishes, including the two-banded seabream (*Diplodus vulgaris*), white seabream (*Diplodus sargus*), European bass (*Dicentrarchus labrax*), Black goby (*Gobius niger*) and thinlip mullet (*Liza ramada*). In these species, both oxidative stress biomarkers and thermal stress biomarkers were increased in response to raised temperatures [3]. Temperature fluctuations can affect a wide range of physiological functions in animals. Long-term exposure to unsuitable but sublethal temperatures may cause suppression of immune response, imbalance in metabolism and accumulation of free radicals, which may then affect growth or reproduction (reviewed in [4–7]). As such, the effects of thermal perturbations are well known in the aquaculture industry; nevertheless, the long-term effects of thermal pollution on wild fish populations have been scarcely documented.

Kuosheng Nuclear Power Plant (NPPII), the largest nuclear power plant in Taiwan, was built in the early 1980’s. Due to a defect in its design, hot effluent water accumulates in the outlet bay (25°12'29.7"N 121°39'46.9"E), creating a thermal plume with high temperature (S1 Fig). In the outlet bay, malformations have been observed in two fish species, the thornfish (*Terapon jarbua*) and largescale mullet (*Planiliza macrolepi*s). The malformed fish are mostly juvenile and are distinct because of a bent backbone (S2 Fig). In some extreme cases, the vertebral columns of the fish may even be wavy. In other cases where similar deformities were observed, many causal factors have been identified, including parasite infection [8], heavy metals [9] or other toxicants [10], radiation [11], and thermal pollution (reviewed in [12]). In the case of NPPII, high temperature discharge-induced thermal pollution is the only one of these factors that has been observed, and it is clear that thermal pollution alone is sufficient to cause skeletal deformities. For example, it has been demonstrated that in the absence of vitamin C supplementation, 88% of thornfish kept in high water temperature (>36°C) for more than 12 weeks exhibited obvious axial deformations [13].

Malformed fishes are widespread in the outlet bay of NPPII during the summer months, especially when the water temperature is above 37°C. The frequency of fish deformations then decreases gradually in the cooler seasons (S1 Table) (reviewed in [13]). Although it is known that the appearance and decline of malformed fish might vary by 1–2 months over the years, the threshold for temperatures that causes deformations has not been previously investigated. In addition, it has been unclear if the malformed juveniles are able to recover during the cooler period, or if their fitness is persistently reduced in subsequent life stages, even after the thermal pollution is mitigated by cooler ambient temperatures. The aims of this study were to determine the threshold temperature and critical exposure time for malformations in the vertebral column of juvenile largescale mullet. Moreover, we aimed to test whether the vertebral deformation could be reversed by transferring fish to lower temperatures. To quantify vertebral deformations, the vertebrae bending index (VBI) was measured from digital X-ray images, which is a far more precise and reliable method than visual inspection used in the earlier studies.

## Materials and methods

### Experimental animals

Juvenile *P*. *macrolepi*s were collected with fish traps near the estuary of Pao-Li creek (22°03'22.7"N 120°42'16.3"E) in southern Taiwan. According to the administering authority, the Pingtung County Marine and Fisheries Management Office, this site is not a protected area and no permission is required for catch. Additionally, there were no endangered or protected species involved in the study. For the study, approximately 4000 juveniles of 1.0–1.5 cm length (standard length; SL) were transferred to the Marine Research Station of Academia Sinica, Yilan, Taiwan, on 23 January 2017. The experiments were performed indoors with a natural photoperiod and air conditioning that kept the room temperature below 26°C. Before the experiments, all fish were kept in a 2-ton circular fiber-reinforced plastic (FRP) tanks for 2 weeks. During this acclimation period, unhealthy fish were removed. Throughout the experiments, fish were fed once per day with commercial fish food (universal pellet, United Aquaculture Feeds, Taiwan) *ad libitum*, without supplementation. All animal care and experiments followed protocols approved by the Institutional Animal Care and Use Committee (IACUC) of National Taiwan Ocean University.

### Experiment 1 (thermal treatment)

For thermal perturbation experiments, juveniles were kept in fifteen 200-L glass aquaria, which were filled with aerated and filtered water. In addition, the aquaria were injected with 0.6 L/min running fresh seawater to keep nitrite salts at a low level. The tops of all aquaria were covered with a piece of 1-cm thick styrofoam. In the beginning of the experiment, each aquarium contained approximately 100 fish, and deceased fish were removed when they were observed.

The temperature of each aquarium was controlled by a heater/circulating module removed from a thermostatic water bath (BH-230D, Yihder co., Ltd, Taiwan). The temperatures of the aquaria were set to be 26°C (control), 30°C, 32°C, 34°C or 36°C. Three aquaria were used for each thermal group. The actual temperature of each aquarium was measured once per day.

Before the treatment, 15 fish were randomly selected to provide an initial control. Then the temperature of the aquarium was adjusted slowly (approximately 2°C per day) to reach the set temperature. During the four-week thermal treatment, ten fish were sampled from each aquarium each week. Including the initial control group, 615 fish (4 weeks × 3 tanks × 5 temperature groups × 10 fish + 15 initial controls) were used for morphological analyses.

### Experiment 2 (recovery)

Juveniles were divided into a 2-ton FRP tank (approximately 2000 fish) and a 200-L glass aquarium (approximately 100 fish) with 2 L/min or 0.6 L/min running fresh seawater. The water temperature of the aquaria was maintained at 26°C (control) by a heater/circulating module, but the seawater in the FRP tank was heated by immersion of a titanium heater (2000W/220V E01C08, Chuan Kuan Enterprise Co., Ltd. Taiwan) adjusted to 36°C by a mechanical thermal start (20A/250V TS-050S, Rainbow Electronics, Co., Ltd. Taiwan). The heat-treatment stage of the experiment lasted for 6 weeks.

At the end of heat-treatment stage, 15 fish were randomly selected from the treatment group (FRP tank) or control group (glass aquaria), and the remaining fish in the heat-treatment group were divided into twelve 200-L glass aquaria (approximately 100 fish each). During the recovery stage, the water temperature of the aquaria was set at 26°C (six aquaria) or 36°C (six aquaria). Fish from all groups, including control (n = 12), were sampled randomly on the fourth (n = 10 from each aquarium, total n = 60 for each group) and eighth week (n = 10 from each aquarium, total n = 60 for each group) of the recovery stage.

### Morphological analysis

**Fig 1:**
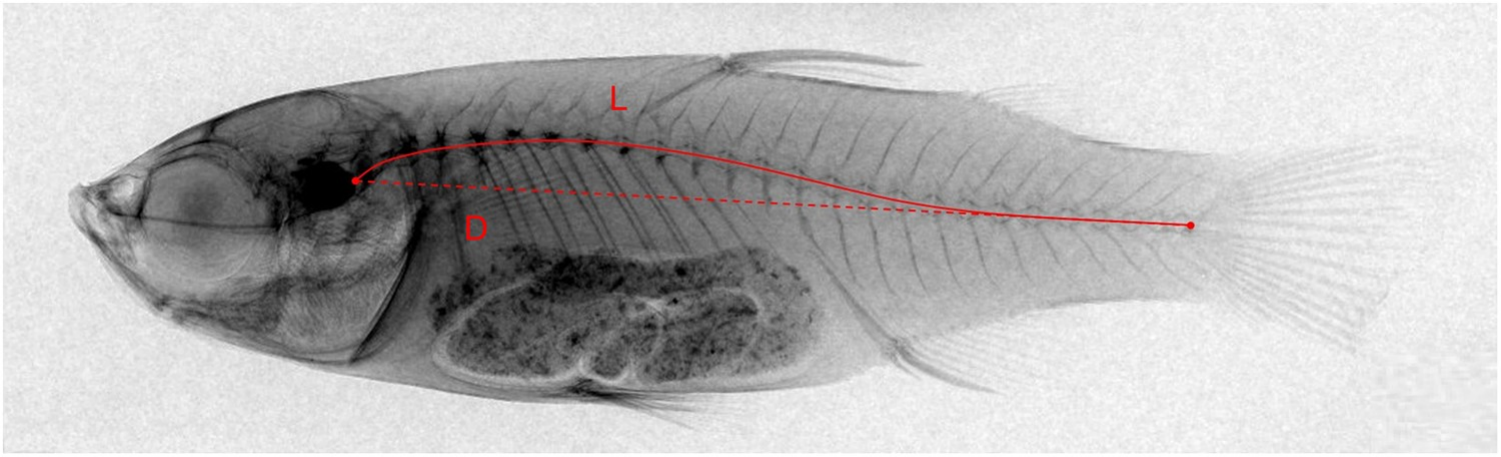
Vertebrae bending index measurement. The vertebrae bending index (VBI) = curve length of vertebral column (L, red solid line) / linear distance between the first vertebrae and urostyle (D, red dotted line).

Before inspection, sampled fish were fixed in 75% EtOH after being euthanized with 0.025% buffered MS-222 (Ethyl 3-aminobenzoate methanesulfonic acid salt, Sigma-Aldrich, US.) solution. A digital CMOS X-ray detector (Model 2315, Dexela, UK) with a custom made emission source set at 45 kV / 120mA with 2.5 sec of exposure, was used to inspect a lateral view of the vertebrae. Using ImageJ (1.05b) software, the curve length of the vertebral column (L) and linear distance between the first vertebrae and urostyle (D) were measured as scaled by a piece of lead wire that is 1 cm in length and 0.3 mm in diameter (Fig 1). Since fish with thermal-induced malformations (mostly categorized as lordosis or kyphosis) typically exhibit longer curve length than linear length of the vertebral column [13] (Figure B in S2 Fig), the vertebral deformations were quantified by the vertebrae bending index (VBI). The VBI was calculated as the vertebral curve length (L) divided by vertebral linear length (D).

### Statistics

Using SPSS v. 19, data from two groups were compared by Mann–Whitney non-parametrical *U*-tests. Multiple group comparisons were performed by the one-way Kruskal–Wallis test, followed by *post hoc* multiple comparisons with Dunn’s test [14, 15].

## Results

The standard lengths and mortalities of the different groups are shown in Tables 1 and 2. In Experiment 1, the lengths of the initial control group were lower than any other group (*p* < 0.05), but no significant differences were found between different temperature treatments during the experimental period (Table 1). In Experiment 2, the standard length of fish that were continuously exposed to 36°C was lower than 26°C controls, but the length of the fish that were switched from 36°C to 26°C showed no difference compared to the controls (Table 2).

**Table 1:** Effects of thermal treatments on the body lengths and mortality in experiment 1.

Groups	Temperature (measured)	Mortality (overall)	SL (cm)
Initial control	-	-	1.35 ± 0.08 *
Control (26°C)	25.8 ± 0.2 °C	0.6 ± 0.5%	2.13 ± 0.13
30°C	30.4 ± 0.3 °C	0% ^a^	2.11 ± 0.10
32°C	32.3 ± 0.2 °C	1.5 ± 1.5%	2.10 ± 0.09
34°C	34.0 ± 0.3 °C	6.8 ± 2.7%	2.09 ± 0.10
36°C	35.9 ± 0.3 °C	31.6 ± 8.3% *	1.98 ± 0.25

Except for the initial control, the standard length (SL) indicates the length of the fish at week 4 (Mean ± SD; *p < 0.05)

**Table 2:** Effects of thermal treatments on the body lengths and mortality in experiment 2.

Groups	Temperature (measured)	Mortality (overall)	SL (cm)
Thermal treated (6 w 36 °C)	36.1 ± 0.2 °C (heat-treatment stage)	-	2.19 ± 0.19 *
Control (6 + 8 w 26°C)	26.1 ± 0.2 °C (recovery stage)	3.2 ± 1.5%	2.83 ± 0.22
Recovery (6 w 36°C + 8 w 26°C)	25.9 ± 0.3 °C (recovery stage)	7.4 ± 2.8%	2.74 ± 0.15
Negative control (6 w + 8 w 36°C)	36.2 ± 0.1 °C (recovery stage)	61.4 ± 15.1% *	2.53 ± 0.18

Except for the initial control, the standard length (SL) indicates the length of the fish after 8 weeks of recovery. (Mean ± SD; *p < 0.05)

### Experiment 1 (thermal treatment)

**Fig 2:**
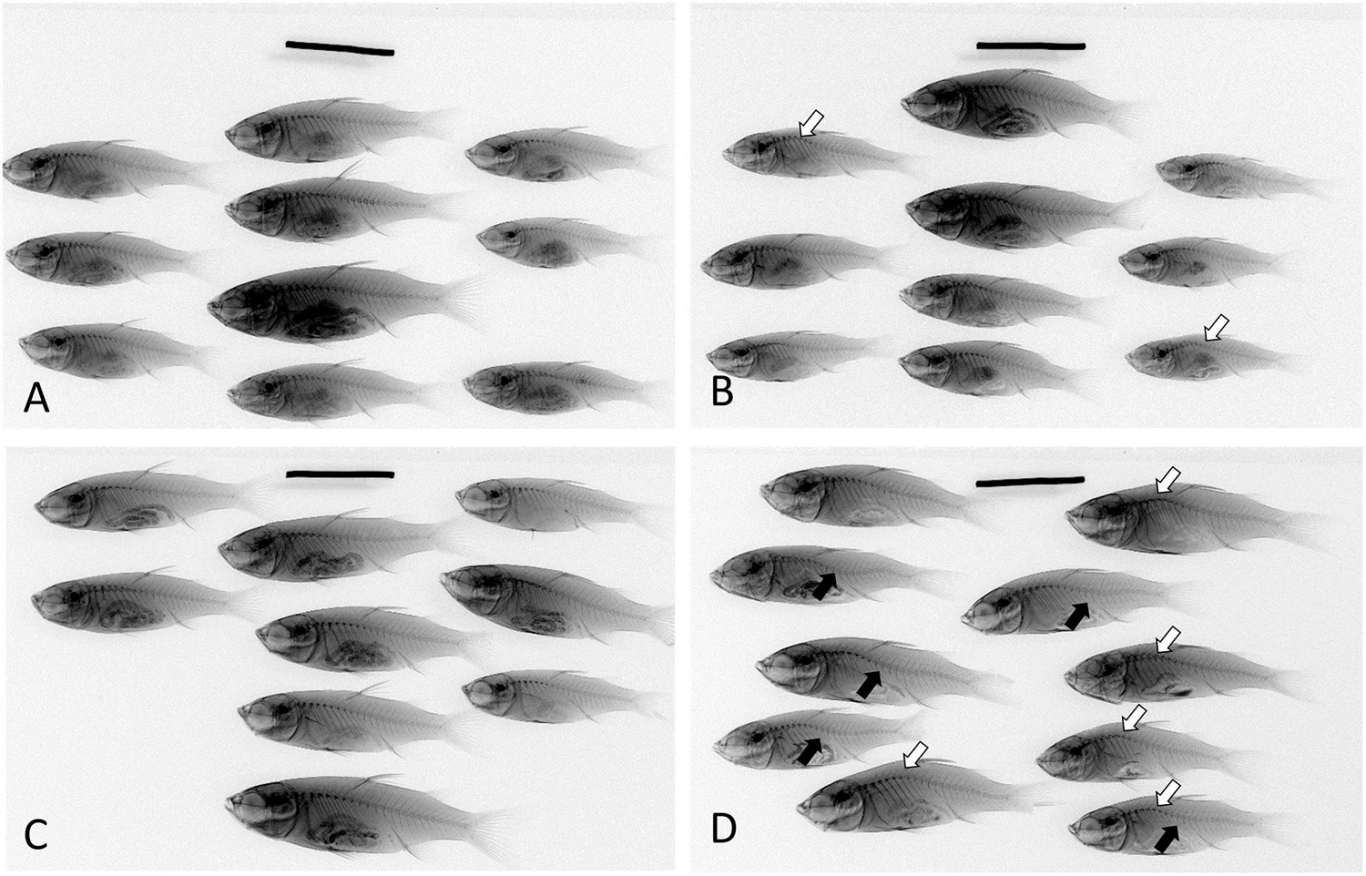
Lateral X-ray views of largescale mullet in Experiment 1. Fish were maintained for (A) 2 weeks at 26°C, (B) 2 weeks at 36°C, (C) 4 weeks at 26°C, or (D) 4 weeks at 36°C prior to X-ray. Open arrows indicate kyphosis, and solid arrows indicate lordosis. Scale bar, 10 mm.

**Fig 3:**
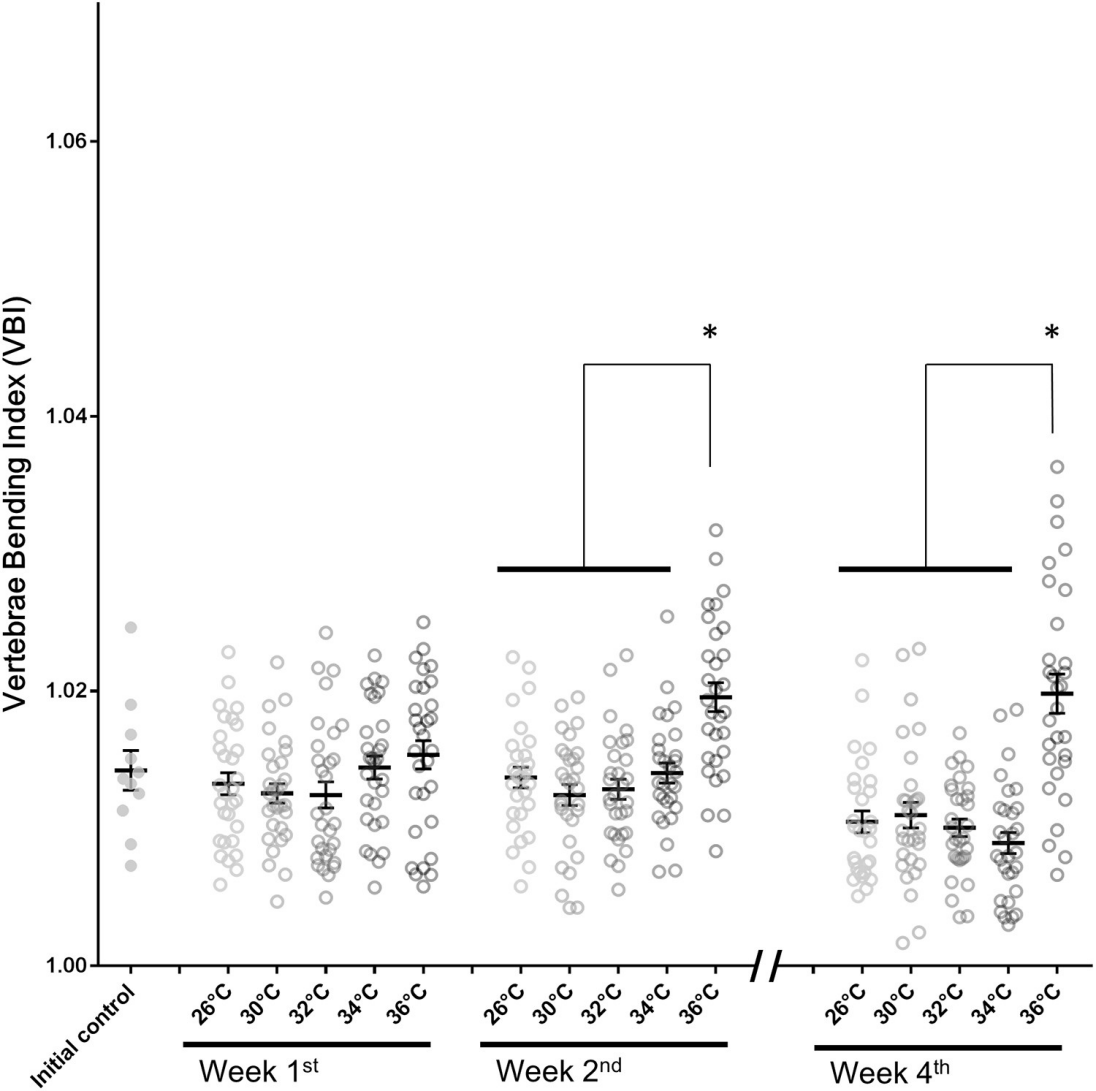
Vertebrae bending index (VBI) of fish kept under different temperatures in Experiment 1. Mean ± SEM, (*p < 0.05).

After 4 weeks of thermal treatment, mortality rates were comparatively low in groups kept at or below 32°C. However, mortality rates were elevated at 34°C and 36°C (Table 1). Additionally, X-ray images clearly showed malformations in the vertebral columns of fish exposed to 36°C for 4 weeks (Fig 2). Compared to the initial control and the 26°C control groups, the 36°C-treated group exhibited clear kyphosis of the spinal column at vertebrae VII-X, with lordosis at vertebrae XV-XVIII (Fig 2). This characteristic malformation could be observed during the last (fourth) week of treatment, but was not readily distinguishable at earlier time points. The malformation was quantified by calculating the VBI for each fish. Differences in VBI were not observed in fish kept at 30°C, 32°C or 34°C at any time point compared to 26°C controls. However, keeping fish at 36°C significantly increased the VBI at week 2 and week 4 (Fig 3).

### Experiment 2 (recovery)

**Fig 4:**
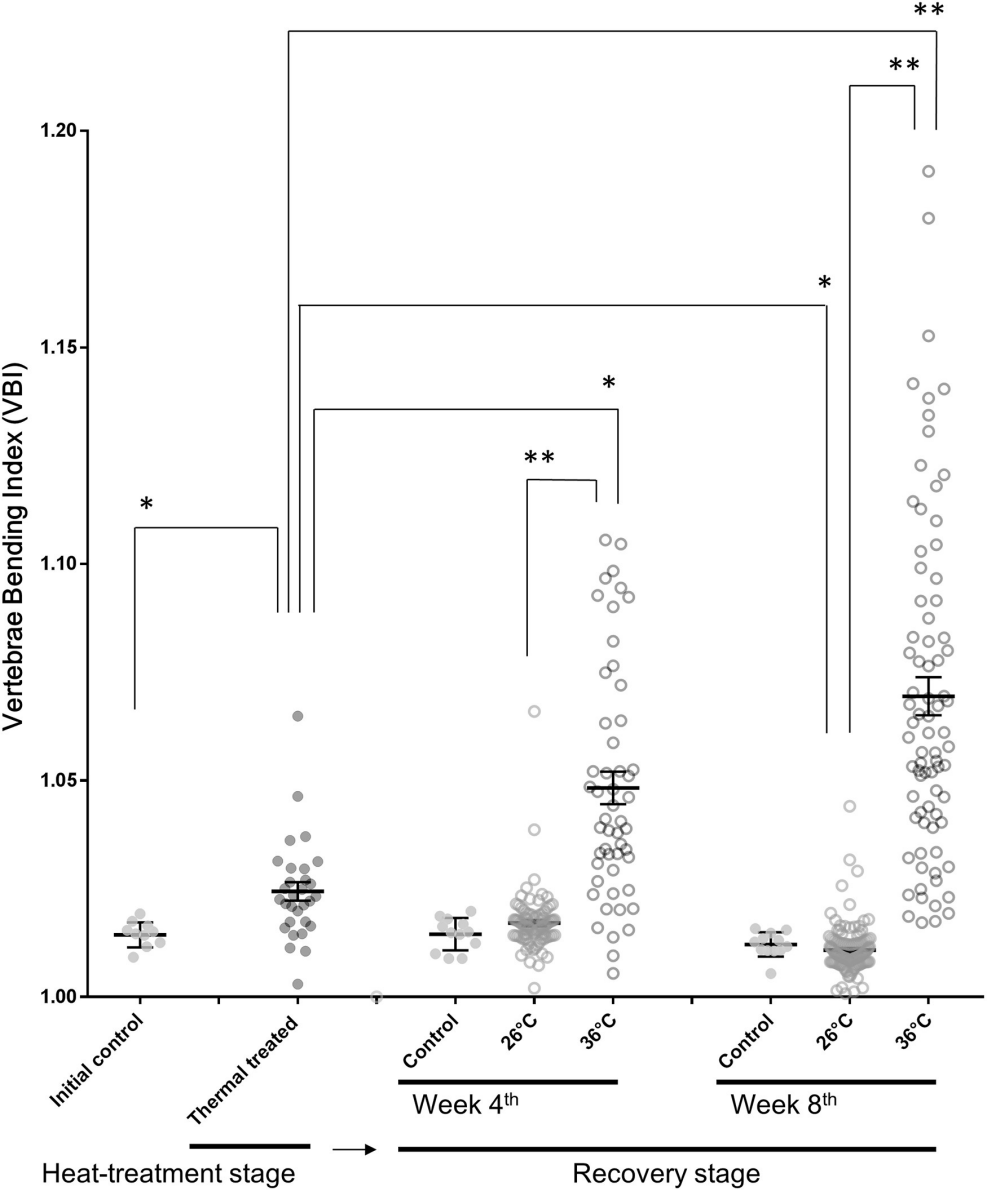
Vertebrae bending index (VBI) in fish kept at 36°C for 6 weeks (heat-treatment stage), followed by 4 or 8 weeks of 36°C or 26°C (recovery stage) in Experiment 2. Control groups were maintained at 26°C for both the heat-treatment and recovery stages. Mean ± SEM, (*p < 0.05).

**Fig 5:**
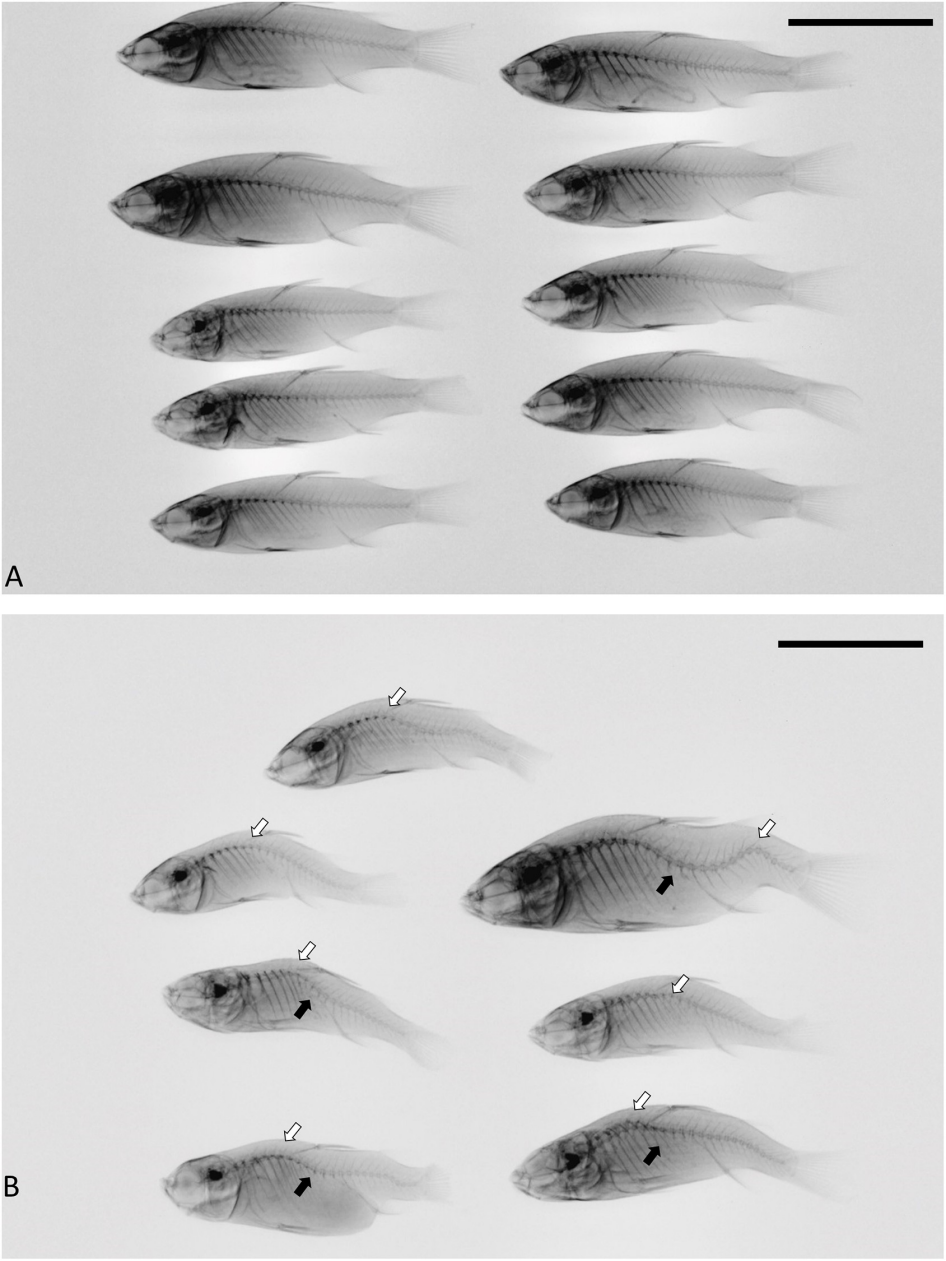
Lateral X-ray views of largescale mullet in Experiment 2. (A) Fish were transferred to 26°C after the heat-treatment stage, or (B) exposure to 36°C was continued for another 8 weeks. Open arrows indicate kyphosis, and solid arrows indicate lordosis. Scale bar, 10 mm.

In the 8-week-recovery experiment, the group maintained at 36°C for the entire duration exhibited a mortality rate of more than 50% (Table 1); however, this high mortality did not occur in either group that was kept at 26°C during the recovery period. After the 6-week heat-treatment stage, the VBI of 36°C-treated fish was higher than initial controls, and this increase was exacerbated in fish that were maintained at 36°C during the recovery stage. Conversely, by the end of the recovery stage, there was no difference in VBI between the 26°C recovery group and the controls that were never exposed to heat treatment (Fig 4). In addition, there was no other apparent malformation in the vertebral columns of fish in the 26°C recovery group (Fig 5A). On the other hand, fish that were maintained at 36°C during the recovery stage exhibited obvious kyphosis and lordosis in the vertebral columns. Abnormal and excessive convex curvatures of the spine typically occurred between vertebrae VII-X and XV-XX (Fig 5B).

## Discussion

Skeletal deformities often reduce the fitness of an individual, potentially by lowering mobility to increase risk of predation. This reduced fitness of malformed individuals explains why malformations are relatively rare in the adults of a wild population. Nevertheless, axial deformations in fish can be found in both wild-born and hatchery-reared larvae/juveniles [16]. Such deformities may result from malnutrition, genetic disease or environmental factors (reviewed in [17]). In a wild population, occurrence of vertebral malformation may occur in various species and populations at low rates. For example, 0.3–0.5% of Atlantic menhaden (*Brevoortia tyrannus*) juveniles sampled from the estuaries between Florida and Massachusetts exhibited malformations [18]. Deformations were also found in 0.5% of star snapper (*Lutjanus stellatus*) and 0.4% of Russell’s snapper (*L*. *russelli*) in populations found off the coast of Miyazaki, Japan [19]. The rate of malformation was reported to be 1–4% in wild gilthead seabream (*Sparus aurata*) post larvae [16, 20], but deformed larvae were extremely rare in red seabream (*Pagrus major*) (reviewed in [21]). NPPII may be the only site where the rate of malformations in a wild population is reported to depend on the severity of thermal pollution/stress. From 1994 to 2004, the overall malformation rate of caught thornfish was 13.7 ± 2.7% (total 5,512 thornfish), while the rate for mullet was 7.3 ± 2.0% (total 7,712 mullet). In the summer months, the rates were up to 18.7 ± 3.3% for thornfish (3,820 thornfish) and 14.4 ± 1.1% for mullet (4,514 mullet) (S1 Table). However, these rates declined drastically in 2017 and 2018, after both reactors were shut down and the water temperature of the outlet bay returned to background levels. According the data from 2017 and 2018, the current overall malformation rate in mullet from the NPPII site is only about 3.5%, as only seven (90–126 mm) deformed fish were identified by visual inspection from 195 collected specimens. The deformities in these fish probably result from biotic/abiotic factors other than thermal pollution. In another set of juvenile largescale mullet that were collected on February 1, 2018 from an area free from thermal pollution (Pao-Li creek), only two of 500 specimens exhibited obvious malformations in the vertebral column.

Indeed, it has been reported that high ratios (up to 63%) of vertebral deformities are found in two natural mosquito fish (*Gambusia afflnis*) populations, which inhabit a spring pool where the temperature was measured to be 34.5–34.8°C [22]. In an open water system, thermal stress caused by high temperature may drive many fishes away, and keeping them at an appropriate distance that may minimize effects. Hence, long-term exposure to thermal pollution is not commonly observed in the field. However, food sources or shelter in a shallow bay may attract some species with relatively high thermal tolerance. As a result, these fish may be exposed to higher risks of effects from thermal pollution. For instance, more than 200 species of fish have been reported to reside in the NPPII outlet bay in the past 20 years, but only a few species, including the malformed species, inhabit areas nearby the effluent outfall. In our study, we found the threshold temperature that induced malformations in largescale mullet was between 34°C and 36°C, which is more precise than the threshold reported in earlier studies [13, 23]. However, our results also showed that deformations occurred earlier than the onset at 4–8 weeks suggested in a previous study [23]. The 36°C thermal treatment significantly increased VBI within 2 weeks, when no obvious difference could be found by visual inspection. Furthermore, there was a clear-cut effect of vertebral deformation at 36°C, whereas no significant difference in VBI was found at or below 34°C. The threshold temperature could potentially result from limitations in metabolic capacity and/or temperature dependence of enzyme activities (reviewed in [24]).

Long-term exposure to high temperatures reduces growth of fish, possibly due to extra energetic costs [25] or suppression of the somatotropic hormone axis, i.e., GH and IGF-1 (reviewed in [26]). In some cases, the function of the GH-IGF-1 system may lead to retarded skeletal development in juvenile fish, such as pejerrey (*Odontesthes bonariensis*) [27]. However, there is no evidence that the somatotropic hormone system is involved in generating thermal-induced deformations. Instead, these malformations have been suggested to result from vitamin and/or mineral imbalances (reviewed in [17]). Deficiency in vitamin C (ascorbic acid) is one factor that results the skeletal malformations, a phenomenon that is well known in aquaculture [7]. Vitamin C is a co-factor in the enzymatic hydroxylation of proline residues in collagen that is essential for the maintenance of connective tissue in vertebrates (reviewed in [28]). Hence, the degradation of vitamin C under warm conditions reduces the ratio of hydroxyproline/proline, which then causes the development of aberrant skeletal structures and impairs bone growth. In previous studies, 16% of thornfish were deformed when kept in 36°C water for 4 weeks, and 31% of the fish were deformed when the exposure period was extended to 8 weeks. Strikingly, no malformed fish were found when the 36°C-treated fish were supplemented with sufficient vitamin C [23]. On the other hand, when the water temperature dropped below the deformation threshold, the fish may be able to conserve dietary vitamin C [13, 23], slowing the development of further deformation. Apart from vitamin C, other vitamins (retinol, retinyl palmitate, retinyl acetate), phospholipids and peptides are known to modulate skeletal growth [29]. Retinoic acid appears to influence skeletal formation in flounder (*Paralichtys olivaceus*) through Hox genes, sonic hedgehog (shh) signaling and retinoic acid receptor (RAR/RXR) responses [30, 31]. Other possible regulatory mechanisms that govern skeletal formation include bone morphogenetic proteins (BMPs) and related molecules, which cause malformations in the jaw of golden pompano (*Trachinotus ovatus*) when dysregulated [32].

Our results suggested that the juvenile mullets had some capacity to self-repair the heat-induced deformation. In Experiment 2, the VBI of malformed fish returned to control levels after an 8-week period of recovery at 26°C. The recovery from thermal-induced axial malformations has not been previously reported. However, an earlier study did demonstrate that young fish could potentially recover from the teratogenic effects of heavy metals, after the pollution source was removed. Chinese sturgeons (*Acipenser sinensis*) that were exposed to lead pollution beginning at the fertilized-egg stage had clear body axis deformations in the juvenile stage. In a subsequent depuration period, the malformations were found to recover along with the decline of tissue lead concentrations [33]. Intriguingly, the physiological mechanisms underlying how a curved vertebral column could be stretched are unknown. There are several possibilities of mechanisms that may accelerate the turnover or the remodeling of deformed spinal columns. As in mammals, bones in teleost species show an adaptive response to altered mechanical loads [34]. Thus, kyphosis and lordosis may cause imbalances in dorsal tissue tension, leading to abnormal mechanical loading of some parts of the spine. In European bass, lordotic vertebrae increase strain energy due to increased spinal compression, which induces trans-differentiation of osteoblastic cells into chondrocytes and may result in a fast adaptive remodeling [35]. In addition, inflammatory cytokines, prostaglandins or leukotrienes are known to regulate osteoblastic and osteoclastic activities [36]. The acute angle of the curved vertebral column may damage the surrounding soft tissues and cause chronic inflammatory responses that could potentially increase bone resorption in some tissues and promote bone deposition in others, depending on the applied mechanical strain [37]. Moreover, the fish used in the present study were in the exponential growth phase [38]; during this period, fish have naturally higher skeleton resorption and remodeling rates than at the adult stage (reviewed in [39]). Our results showed that bone formation or growth in the largescale mullet allows for recovery of some malformations; nevertheless, it is reasonable to expect that the capacity to recover may depend on the initial body size or the growth rate of the malformed fish.

The possibility of tolerance or recovery from chronic effects of thermal pollution was considered to be important for aquaculture and marine conservation when the thermal outlet bay was designed. In order to assess the impacts of thermal pollution on fish, Bevelhimer & Bennett [40] proposed a model wherein stress accumulation occurs above a threshold temperature at a rate dependent on the degree to which the threshold is exceeded, and thermal stress recovery may occur when temperatures drop below the threshold. It is common for many thermally polluted areas to experience daily or seasonal fluctuations [40]. When the NPPII site was operational, summer water temperatures were usually above 36°C, which is higher than the deformation threshold, but below 26°C from October to June. Hence, the fishes in the areas may have experienced different thermal impacts depending on their seasonal activities. For example, juvenile fish from species that settle in the area late in the summer may experience a shorter exposure time and longer recovery time than those that settle earlier.

The impact of power plants on the thermal characteristics of aquatic ecosystems has been a concern for decades. Temperature criteria for effluents or limitations on temperature differences between intake and outlet areas are critical to minimize thermal pollution. Moreover, the physical design of the artificial structure may also influence the impacts of thermal pollution. For example, the thermal discharges from other nuclear or fossil power plants in Taiwan are flushed into the open sea and have no harbor-like structure that accommodates fish for long periods. In addition to the accurate and timely monitoring of fish assemblage, our study suggests that the physiological differences among the resident species, including temperature tolerance, life history and breeding season should be considered to precisely mitigate the potential environmental impacts of a thermal pollution source.

## Conclusions

The waters near NPPII in Taiwan are one of the rare sites where thermal pollution has increased the proportion of malformed fish in a wild population. In this study, we demonstrated that VBI is an effective parameter to measure spinal malformations. As such, VBI provided a sensitive and reliable measurement to assess thermal threshold and exposure time. Furthermore, our results suggested that the juvenile mullets have some capacity to self-repair heat-induced deformations. However, the ability to recover may depend on the initial body size or growth rate of the malformed fish.

## Supporting information

S1 Table Characterization of the NPPII site. 1) The average water temperature of the site (1997–2004) is shown. Red columns indicate temperatures above 36°C. 2) The malformation rate of fish caught in the waters near the NPPII bay (1997–2004). N.A. indicates a month when there was no catch. Largescale mullet (*P*. *macrolepis*), thornfish (*T*. *jarbua*), milkfish (*Chanos chanos*) and flathead grey mullet (*Mugil cephalus*). The data in this figure were provided by the Taipower company and are derived from a long-term monitoring project.
(TIF)

S1 Fig Diagram of the outlet bay of NPPII. A dotted line shows the embankment before reconstruction (1994/Mar), and the red circle indicates the hot spot where malformed fishes were often observed.
(TIF)

S2 Fig An example of malformed fish. Bright-field (A) and X-ray (B) images of fish collected from the outlet bay of NPPII on February 23, 2016. One malformed largescale mullet (arrow) is identified in the group.
(TIF)

## References

[1] Clark JR. Thermal pollution and aquatic life. Sci Am. 1969; 220(3): 18–27.

[2] Cooke SJ, Schreer JF. Environmental monitoring using physiological telemetry–a case study examining common carp responses to thermal pollution in a coal-fired generating station effluent. Water Air Soil Pollut. 2003; 142(1–4): 113–136.

[3] Madeira D, Narciso L, Cabral HN, Vinagre C, Diniz MS. Influence of temperature in thermal and oxidative stress responses in estuarine fish. Comp Biochem Physiol A Mol Integr Physiol. 2013; 166(2): 237–243. 10.1016/j.cbpa.2013.06.008 23774589 10.1016/j.cbpa.2013.06.008

[4] Le Morvan C, Troutaud D, Deschaux P. Differential effects of temperature on specific and nonspecific immune defences in fish. J Exp Biol. 1998; 201(2): 165–168. 9405298 10.1242/jeb.201.2.165

[5] Tomanek L. Variation in the heat shock response and its implication for predicting the effect of global climate change on species’ biogeographical distribution ranges and metabolic costs. J Exp Biol. 2010; 213(6): 971–979. 10.1242/jeb.038034 20190122 10.1242/jeb.038034

[6] Kingsolver JG, Woods HA. Beyond thermal performance curves: modeling time-dependent effects of thermal stress on ectotherm growth rates. Am Nat. 2016; 187(3): 283–294. 10.1086/684786 26913942 10.1086/684786

[7] Dadras H, Dzyuba B, Cosson J, Golpour A, Siddique MAM, Linhart O. Effect of water temperature on the physiology of fish spermatozoon function: a brief review. Aquacult Res. 2017; 48(3): 729–740.

[8] Kelly DW, Thomas H, Thieltges DW, Poulin R, Tompkins DM. Trematode infection causes malformations and population effects in a declining New Zealand fish. J Animal Ecol. 2010; 79(2): 445–452. 10.1111/j.1365-2656.2009.01636.x 19886894

[9] Sfakianakis DG, Renieri E, Kentouri M, Tsatsakis AM. Effect of heavy metals on fish larvae deformities: a review. Environ Res. 2015; 137: 246–255. 10.1016/j.envres.2014.12.014 25594493 10.1016/j.envres.2014.12.014

[10] Yershov PN. The vertebral abnormalities in eelpout *Zoarces viviparus* (Linnaeus, 1758) (Pisces, Zoarcidae). Proceedings ZIN. 2008; 312(1–2): 74–82.

[11] Bogutskaya NG, Zuykov MA, Naseka AM, Anderson EB. Normal axial skeleton structure in common roach *Rutilus rutilus* (Actinopterygii: Cyprinidae) and malformations due to radiation contamination in the area of the Mayak (Chelyabinsk Province, Russia) nuclear plant. J Fish Biol. 2011; 79(4): 991–1016. 10.1111/j.1095-8649.2011.03078.x 21967586 10.1111/j.1095-8649.2011.03078.x

[12] Wetang’ula GN. Assessment of geothermal wastewater disposal effects: case studies: Nesjavellir (Iceland) and Olkaria (Kenya) fields. M.Sc. Thesis, The United Nations University, Geothermal Training Programme. 2004. http://www.os.is/gogn/flytja/JHS-Skjol/Yearbook2004/02GabrielMSc.pdf

[13] Hwang DF, Chien LT, Shao KT, Jeng SS. Levels of heavy metals and vitamin C in deformed thornfish found in thermal waters and effect of vitamin C on deformation of thornfish. Fish Res. 1998; 64(2): 291–294.

[14] Folkvord A. Growth, survival and cannibalism of cod juveniles (*Gadus morhua*): effects of feed type, starvation and fish size. Aquaculture. 1991; 97(1): 41–59.

[15] Lewis LM, Lall SP, Witten PE. Morphological descriptions of the early stages of spine and vertebral development in hatchery-reared larval and juvenile Atlantic halibut (*Hippoglossus hippoglossus*). Aquaculture. 2004; 241(1–4): 47–59.

[16] Boglione C, Gagliardi F, Scardi M, Cataudella S. Skeletal descriptors and quality assessment in larvae and post-larvae of wild-caught and hatchery-reared gilthead sea bream (*Sparus aurata* L. 1758). Aquaculture. 2001; 192(1): 1–22.

[17] Brown CL, Power DM, Núñez JM. Disorders of Development in Fish In: Leatherland JF, Woo PTK, editors. Fish Diseases and Disorders: Non-infectious Disorders. CABI Publishing, Wallingford, UK; 2010 pp. 166–181.

[18] Kroger RL, Guthrie JF. Incidence of crooked vertebral columns in juvenile Atlantie menhaden, *Brevoortia tyrannus*. Chesapeake Sci. 1971; 12: 276.

[19] Endo M, Uehara K, Iwatsuki Y. Body anomalies due to spinal curvature in two species of snappers *Lutjanus stellatus* and *L*. *russelli* from the coast off Miyazaki, Southern Japan. Japan J. Ichthyol. 1994; 41(1): 76–79.

[20] Francescon A, Freddi A, Barbaro A, Giavenni R. Daurade *Sparus aurata* L. reproduite artificiellement et daurade sauvage. Expériences paralleles en diverses conditions d'élevage. Aquaculture. 1988; 72(3–4): 273–285.

[21] Al-Mamry J. M., Jawad L. A., Al-Rasady I. H., & Al-Habsi S. H. (2010, 1). First record of dorsal and anal fin deformities in silver pomfrets, *Pampus argenteus* (Stromateidae, Actinopterygii)/Primer registro de deformidades en las aletas dorsal y anal de la palometa plateada, *Pampus argenteus* (Stromateidae, Actinopterygii). An Biol. 2010; 32: 73–77.

[22] Hubbs C. High incidence of vertebral deformities in two natural populations of fishes inhabiting warm spring. Ecology. 1959; 40: 154.

[23] Chien LT, Hwang DF, Jeng SS. Effect of thermal stress on dietary requirement of vitamin C in Thornfish *Terapon jarbua*. Fish Sci. 1999; 65(5): 731–735.

[24] Darias MJ, Mazurais D, Koumoundouros G, Cahu CL, Zambonino-Infante JL. Overview of vitamin D and C requirements in fish and their influence on the skeletal system. Aquaculture. 2011; 315(1–2): 49–60.

[25] Houde ED. Comparative growth, mortality, and energetics of marine fish larvae: temperature and implied latitudinal effects. Fish Bull. 1989; 87(3): 471–495.

[26] Gabillard JC, Weil C, Rescan PY, Navarro I, Gutierrez J, Le Bail PY. Does the GH/IGF system mediate the effect of water temperature on fish growth? A review. Cybium. 2005; 29(2): 107–117.

[27] Gómez-Requeni P, Kraemer MN, Canosa LF. Regulation of somatic growth and gene expression of the GH–IGF system and PRP-PACAP by dietary lipid level in early juveniles of a teleost fish, the pejerrey (*Odontesthes bonariensis*). J Comp Physiol B. 2012; 182(4): 517–530. 10.1007/s00360-011-0640-9 22227923 10.1007/s00360-011-0640-9

[28] Englard S, Seifter S. The biochemical functions of ascorbic acid. Annu Rev Nutr. 1986; 6(1): 365–406. 3015170 10.1146/annurev.nu.06.070186.002053

[29] Cahu C, Infante JZ, Takeuchi T. Nutritional components affecting skeletal development in fish larvae. Aquaculture 2003; 227(1–4): 245–258.

[30] Suzuki T, Oohara I, Kurokawa T, Hoxd-4 expression during pharyngeal arch development in flounder (*Paralichtys olivaceus*) embryos and effects of retinoic acid on expression. Zool Sci 1998; 15: 57–67. 10.2108/zsj.15.57 9615618 10.2108/zsj.15.57

[31] Suzuki T, Oohara I, Kurokawa T. Retinoic acid given at late embryonic stage depresses sonic hedgehog and Hoxd-4 expression in the pharyngeal area and induces skeletal malformation in flounder (*Paralichtys olivaceus*) embryos. Develop. Growth Differ. 1999; 41: 143–152. 10.1046/j.1440-169x.1999.00420.x 10223710

[32] Ma Z, Zhang N, Qin JG, Fu M, Jiang S. Water temperature induces jaw deformity and bone morphogenetic proteins (BMPs) gene expression in golden pompano *Trachinotus ovatus* larvae. Springerplus. 2016; 5(1): 1475 10.1186/s40064-016-3142-0 27652050 10.1186/s40064-016-3142-0 PMC5010545

[33] Hou JL, Zhuang P, Zhang LZ, Feng L, Zhang T, Liu JY, Feng GP. Morphological deformities and recovery, accumulation and elimination of lead in body tissues of Chinese sturgeon, *Acipenser sinensis*, early life stages: a laboratory study. J Appl Ichthyol. 2011; 27(2): 514–519.

[34] Fiaz AW, Van Leeuwen JL, Kranenbarg S. Phenotypic plasticity and mechano‐transduction in the teleost skeleton. J. Appl. Ichthyol. 2010; 26(2): 289–293.

[35] Kranenbarg S, Van Cleynenbreugel T, Schipper H, Van Leeuwen J. Adaptive bone formation in acellular vertebrae of sea bass (*Dicentrarchus labrax* L.). J. Exp. Biol. 2005; 208(18): 3493–3502. 16155222 10.1242/jeb.01808

[36] Gil‐Martens L. Inflammation as a potential risk factor for spinal deformities in farmed Atlantic salmon (*Salmo salar* L.). J. Appl. Ichthyol. 2001; 26(2): 350–354.

[37] Robling AG, Castillo AB, Turner CH. Biomechanical and molecular regulation of bone remodeling. Annu. Rev. Biomed. Eng. 2006; 8: 455–498. 10.1146/annurev.bioeng.8.061505.095721 16834564 10.1146/annurev.bioeng.8.061505.095721

[38] Caona L. Effects of salinity on the habitat selection and growth performance of Mediterranean flathead grey mullet Mugil cephalus (Osteichthyes, Mugilidae). Estuar Coast Shelf Sci. 2000; 50(5): 727–737.

[39] Lall SP, Lewis-McCrea LM. Role of nutrients in skeletal metabolism and pathology in fish—an overview. Aquaculture. 2007; 267(1–4): 3–19

[40] Bevelhimer M., & Bennett W. (2000). Assessing cumulative thermal stress in fish during chronic intermittent exposure to high temperatures. Environ Sci Policy. 2000; 3: 211–216.

