# Supplementary figures and images for: Largescale mullet (Planiliza macrolepis) can recover from thermal pollution-induced malformations
Source: PLoS One. 2018 Nov 29;13(11):e0208005. doi: 10.1371/journal.pone.0208005 (PMC6264803; doi:10.1371/journal.pone.0208005)

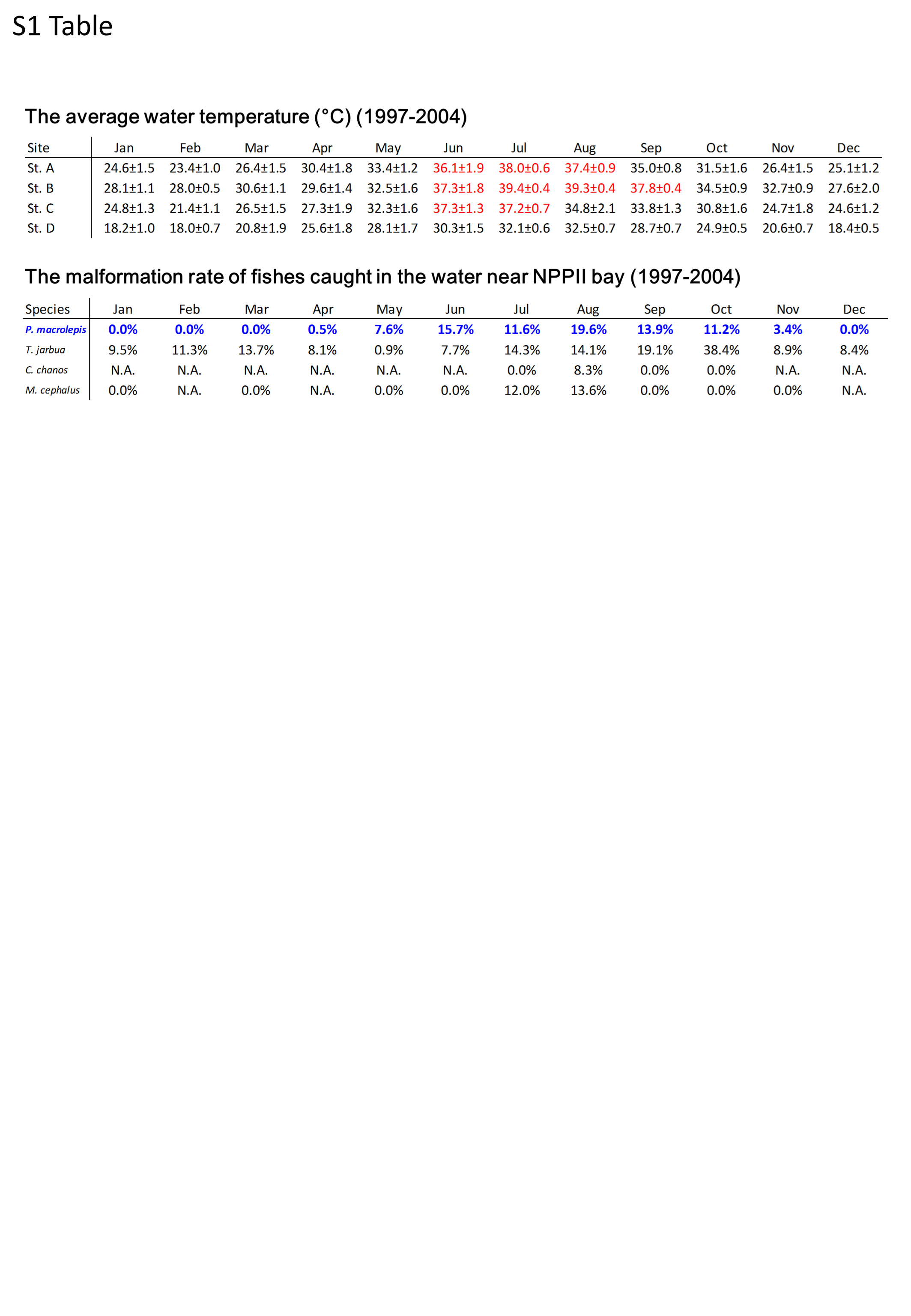

Supplement: S1 Table — 1) The average water temperature of the site (1997–2004) is shown. Red columns indicate temperatures above 36°C. 2) The malformation rate of fish caught in the waters near the NPPII bay (1997–2004). N.A. indicates a month when there was no catch. Largescale mullet (P. macrolepis), thornfish (T. jarbua), milkfish (Chanos chanos) and flathead grey mullet (Mugil cephalus). The data in this figure were provided by the Taipower company and are derived from a long-term monitoring project. (TIF) [file pone.0208005.s001.TIF]

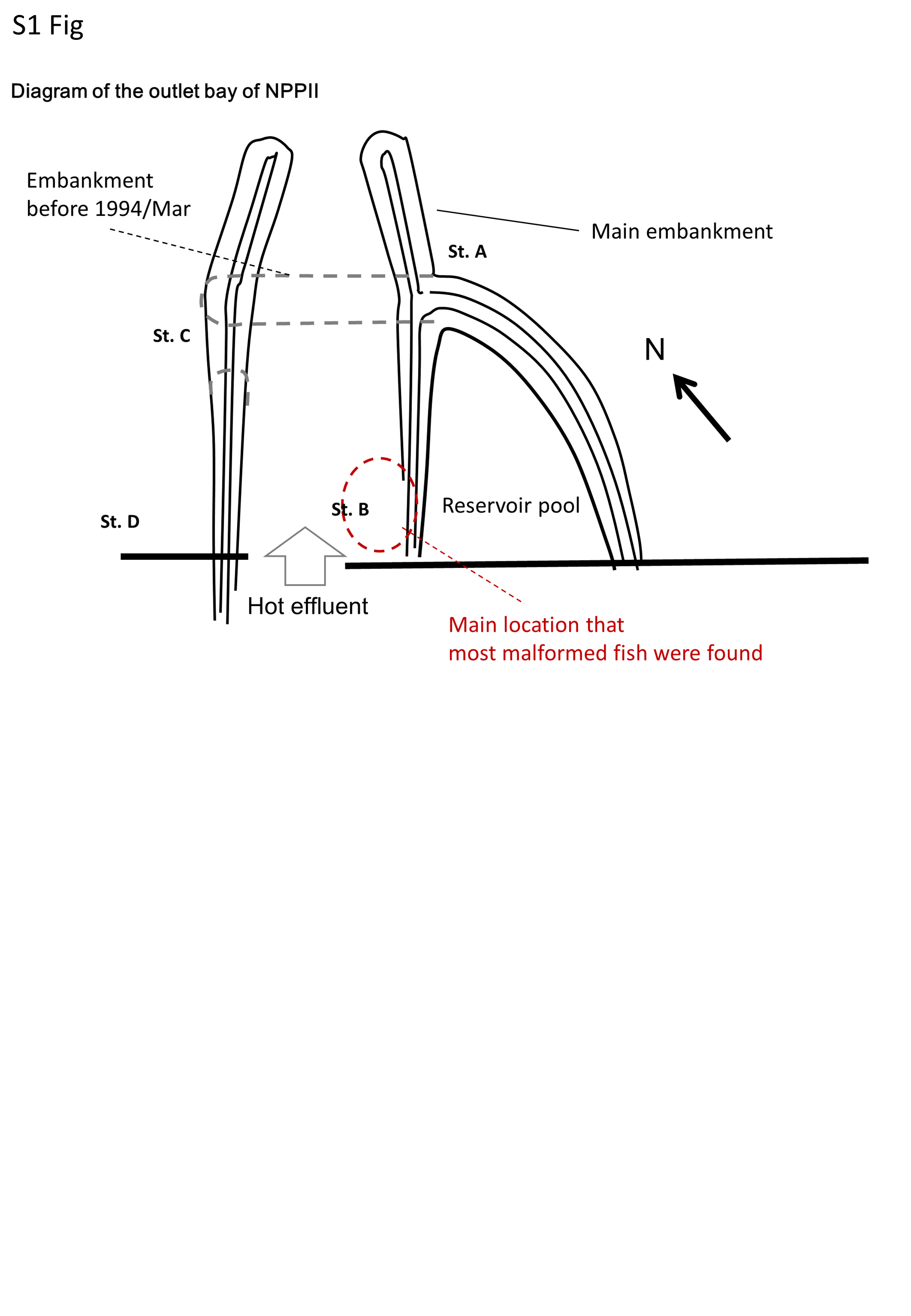

Supplement: S1 Fig — A dotted line shows the embankment before reconstruction (1994/Mar), and the red circle indicates the hot spot where malformed fishes were often observed. (TIF) [file pone.0208005.s002.TIF]

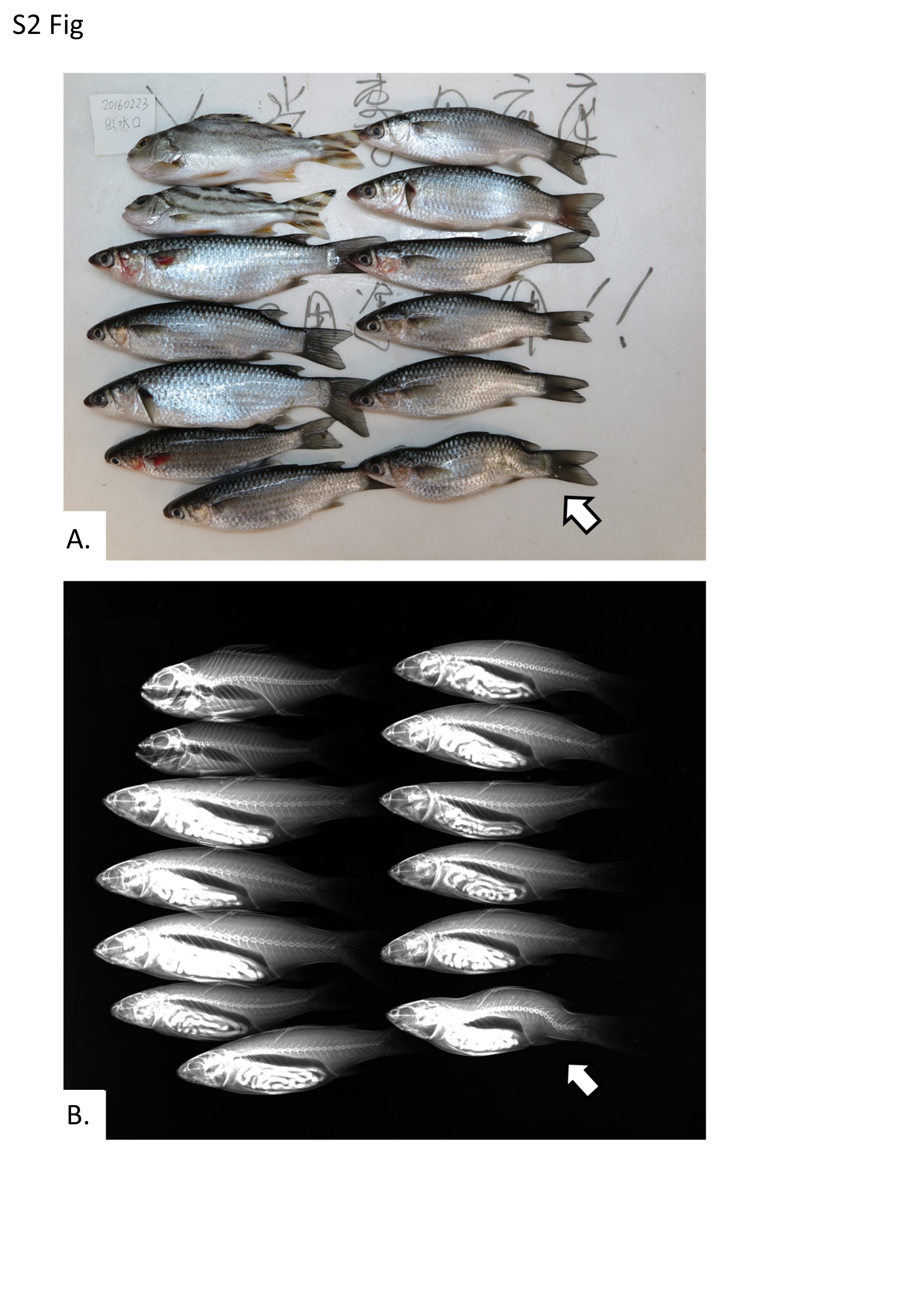

Supplement: S2 Fig — Bright-field (A) and X-ray (B) images of fish collected from the outlet bay of NPPII on February 23, 2016. One malformed largescale mullet (arrow) is identified in the group. (TIF) [file pone.0208005.s003.tif]
